# 

**Published:** 1874-03

**Authors:** 


### CONTENTS:

### ORIGINAL COMMUNICATIONS.

ARTICLE I.—Professional Excellence. By W. W. H. Thackston, M. D., D. D. S. ........ 481

ARTICLE II.—Neuralgia of the Trigeminus. By L. Jacobi, D. D. S. ........ 490

### SELECTED ARTICLES.

ARTICLE III.—Traumatic Phthisis. By C. F. Bevan, M. D ........ 508

ARTICLE IV.—Leeches and How I Manage Them ........ 512

### EDITORIAL, ETC.

The Thirty-fourth Annual Commencement of the Baltimore College of Dental Surgery ........ 517
Character of Amalgams ........ 519
Expansion of the Arch in the Treatment of Irregularity of the Teeth ........ 521
Mississipi Valley and Missouri State Dental Association ........ 522

### MONTHLY SUMMARY.

Transplantation of Teeth ........ 522

Mental Hygiene ........ 523

The New Local Anæsthetic ........ 523

Microscopic Terrors ........ 524

Methylene Ether as an Anæsthetic ........ 524

The Human Body Compared to a Machine ........ 525

Bad Effects of Thumb-sucking ........ 525

Sugar and Magnesia an Antidote to Arsenic ........ 525

Nervous and Sick Headache ........ 526

The Blood Cure ........ 526

Diagnosis of Lipomata ........ 526

Important Point in Treatment of Frozen Limbs ........ 527

Laws of Health ........ 527

Ether Glue ........ 527

Woman Dentists in Egypt ........ 528

Chloroform in Dentistry ........ 528

Persecution of Male Students ........ 528

Treatment of Burns and Scalds ........ 528

Lime Water in Stings of Bees and Wasps ........ 528

